# Pharmaceutical interventions on prescriptions in Norwegian community and hospital pharmacies

**DOI:** 10.1007/s11096-020-01188-w

**Published:** 2020-11-06

**Authors:** Steinar Vik, Pernille Weidemann, Ingrid Elisabeth Mehl Gangås, Stein-Erik Knapstad, Svein Haavik

**Affiliations:** 1 Department of Quality and Development, Hospital Pharmacies Enterprise in Western Norway, Bergen, Norway; 2 Boots Pharmacy Revetal, Wallgreens Boots Alliance, Revetal, Norway; 3 Levanger Hospital Pharmacy, Central Norway Hospital Pharmacy Trust, Levanger, Norway; 4 Centre for Pharmacy, Department of Clinical Science, University of Bergen, Bergen, Norway

**Keywords:** Adverse drug events, Electronic prescriptions, Generic substitution, Pharmacist interventions, Primary care

## Abstract

*Background* Pharmacists in community and hospital pharmacies assess prescriptions to prevent prescription errors and adverse drug events. There are, however, few reports on prevalence of clinical important pharmaceutical interventions for patients located within primary care. *Objective* To study documented pharmaceutical interventions on prescriptions in Norwegian pharmacies for patients located in primary care. *Setting* Data were collected in 11 community pharmacies during a 3 months period in 2016, and the outpatient department of four hospital pharmacies in Norway during a 6 months period of 2018. *Method* Retrospective analysis of electronically documented pharmaceutical interventions on prescriptions for patients located in primary care. *Main outcome measure* The number and classification of pharmaceutical interventions in relation to the total number of prescriptions. *Results* An intervention was documented in 124,178 (45.1%) of the 275,339 prescriptions dispensed during the study period. Interventions of potential clinical importance were performed and documented in 0.8% (2262) of the prescriptions. *Conclusion* A substantial number of pharmaceutical interventions are performed on prescriptions in Norwegian pharmacies after introduction of electronic prescriptions. A potentially clinical important intervention is performed in one of every 125 prescriptions (0.8%). This result indicates that pharmacists at Norwegian pharmacies prevent more than 400,000 prescription errors of potential clinical importance each year.

## Impact on practice

- The introduction of electronic prescriptions (EP) was presumed to increase patient safety and reduce prescription errors.
- After introduction of EP in Norway, the pharmacists are identifying a similar prevalence of prescribing problems needing to be resolved to avoid patient harm or adverse drug events as found in studies undertaken before the introduction of EP.
- By assessing EP during dispensing, the Norwegian pharmacists prevent more than 400,000 prescription errors of potential clinical importance for patients in primary care each year.

## Introduction

The pharmacists have a vital role in preventing adverse drug events (ADE) by detecting prescribing errors of potential clinical importance [1, 2]. ADE represents a substantial burden for the affected patients and the health care system, with an estimated incidence of 10% of all admissions in acute care setting being related to ADE [3]. The Norwegian government initiated a national eHealth system with the aim to increase patient safety including electronic prescribing (EP) in outpatient settings in 2013 and hospitals in 2016 [4].

Both the Norwegian community pharmacies and the hospital pharmacies are using the same computer software to manage prescriptions and document pharmaceutical interventions. All interventions documented in the pharmacy electronic records, are resolved cases where the pharmacist have identified and performed a required intervention.

Previous studies undertaken before the introduction of EP, have shown that pharmacist interventions have been performed in 2–3% of all prescriptions, with a potential clinical important intervention being performed in 0.4–0.5% of all prescriptions [5–9]. The main goals of introducing EP in the Norwegian health care system, was to increase patient safety by eliminating transcriptional errors and reduce the incidence of prescribing errors. The improved quality of prescriptions would reduce the need of pharmaceutical interventions and give the pharmacist an opportunity to increase focus on patient education to further prevent ADE.

### Aim of the study

This paper describes a retrospective study of electronically documented pharmaceutical interventions on prescriptions in Norwegian pharmacies for patients located in primary care after introduction of EP.

### Ethics approval

The regional committee for medical and health research ethics of Western Norway (REC West) confirmed that ethical approval was not needed for this study as it was based on analysis of electronic records without personal identifiable information (2017/1678).

## Method

The study was performed as a two stages retrospective collection of electronically documented pharmaceutical interventions in a total of 15 participating pharmacies. To eliminate bias of the retrieved data, the periods of data collection was prior to the time of inclusion of the pharmacies in the study. The first stage of the data collection was performed by 11 community pharmacies during the last 3 months of 2016. All community pharmacies belonged to the same pharmacy chain, and the inclusion was based on request to the pharmacy chain to contribute with one voluntary participating pharmacy from each geographical region of Norway. Of the 17 possible regions, a total of 11 regions were represented with one participating community pharmacy.

The second stage of the data collection was performed by an identical protocol by four hospital pharmacies with retrospective data collected from the first 6 months of 2018. The hospital pharmacies were situated in the western region of Norway and recruited by request from Hospital Pharmacies West Enterprise, Norway.

The pharmacy manager of each participating pharmacy exported all electronically documented pharmaceutical interventions of the study period to a spread sheet. Columns containing patient and prescriber information were deleted to anonymize the data before sending the data for analysis. A successive number was automatically assigned to each prescription to identify when more than one intervention was undertaken for a single prescription. The pharmacies also reported the total number of prescriptions dispensed in the study period.

The interventions reported by the pharmacies were categorized according to the category selected by the pharmacist in the pharmacy computer software during dispensing of the prescription. When the pharmacists record interventions, they choose the possible cause from a list of options in the pharmacy computer program. Some of the available categories of interventions to choose from, can be considered as interventions of technical nature and others as interventions of potential clinical importance. An example of an intervention of technical nature is change in package size due to shortage or change to a lower cost generic alternative brand due to reimbursement regulations. In Norway, the pharmacists are required by law to offer the generic alternative of lowest cost to the patient, if the prescriber has not restricted a generic substitution on the prescription due to a medical reason like a previously adverse reaction to the generic alternative. If there is an alternative generic drug in the market at a lower price than the prescribed drug, the pharmacy computer program will automatically suggests an intervention to a generic alternative drug of same strength, dosage form and package size. The Norwegian Medicines Agency defines which generic products that are interchangeable based on a set of criteria including equivalent bioavailability. The pharmacist does not have to inform the prescriber when performing an intervention to substitute between such generic alternatives. Generic substitutions by the pharmacist have significant ramifications for the healthcare costs, as government reimbursement systems cover the main costs of the medicines for chronic diseases. An increased pharmacy mark-up for generic products, is the economic incentive to perform such pharmacoeconomic interventions in the pharmacies. Patients may decline the offer of a generic alternative of lower cost, but they must then cover the extra cost unless the doctor has restricted a generic substitution for medical reasons on the prescription.

When the pharmacist makes an intervention of potential clinical importance, the prescriber needs to authorise the change. The available categories of interventions of potential clinical importance in the pharmacy computer software are listed in Table 2.

Some interventions were found to be assigned to a misleading category. Reassignment of these to a more appropriate category was decided after discussion of each case in the research team consisting of two pharmacy students, two senior pharmacist with 15–20 years of experience in dispensing prescriptions, and a professor in pharmacy at University of Bergen.

Fisher´s exact test calculations was performed to assess the statistical significance of the differences between community pharmacies and hospital pharmacies.

## Results

A total of 275,339 prescriptions were dispensed during the two study periods at the 15 participating pharmacies (Table 1). A total of 124,178 (45.1%) of the prescriptions had been subjected to one or more intervention of either technical or pharmacoeconomic nature, or interventions of potential clinical importance. The main causes of interventions were of pharmacoeconomic nature, by substituting from prescribed medication to a generic medication at a lower price. Interventions of pharmacoeconomic nature were performed in 93,576 (33.9%) of the prescriptions, and other interventions of technical nature were performed on 28,340 (10.3%) of the prescriptions. It was found that the pharmacist in 2262 (0.8%) of the cases did perform an intervention of potential clinical importance. Interventions of potential clinical importance was more frequently documented in hospital pharmacies than in community pharmacies (*p* < 0.00001) mainly due to a higher prevalence of interventions of miscellaneous type documented by a free text field due to no suitable category available in the computer software.

**Table 1:** Total number of prescriptions and number of prescriptions with recorded generic switch, logistic switch and Interventions of potential clinical importance (IPCI) during the study period

Pharmacy	Prescriptions	Generic switch	Logistic switch	IPCI
Community	155,772	51,386 (32.9%)	19,843 (12.7%)	740 (0.5%)
Hospital	119,567	42,190 (35.3%)	8497 (7.1%)	1522 (1.3%)
Total	275,339	93,576 (33.9%)	28,340 (10.3%)	2262 (0.8%)

Table 1 shows the distribution of technical interventions like changes of logistical nature or generic substitutions and interventions of potential clinical importance.

Table 2 gives an overview of interventions classified as potential clinical important, arranged by ascending numbers of total incidents. Precautionary issues are overlooked warnings of special cautions when using the medicine for some patient groups. The miscellaneous group represents interventions of potential clinical importance that did not fit into other categories, like changing to smaller tablets due to difficulties in swallowing large tablets.

**Table 2:** The number of interventions recorded in the community and hospital pharmacies

Intervention	Community	Hospital	Total	p*
Precautionary issue	0 (0%)	1 (0.04%)	1 (0.04%)	0.4343
Contraindication	0 (0%)	6 (0.3%)	6 (0.3%)	0.0067
Change due to adverse effect	9 (0.4%)	2 (0.09%)	11 (0.5%)	0.1286
Wrong substance	5 (0.2%)	6 (0.3%)	11 (0.5%)	0.5484
Interaction	2 (0.09%)	15 (0.7%)	17 (0.8%)	0.0002
Change of dosing time or interval	16 (0.7%)	45 (2.0%)	61 (2.7%)	< 0.00001
Too high dose	8 (0.4%)	68 (3.0%)	76 (3.4%)	< 0.00001
Too low dose	30 (1.3%)	116 (5.1%)	146 (6.5%)	< 0.00001
Wrong amount	28 (1.2%)	166 (7.3%)	194 (8.6%)	< 0.00001
Wrong strength	163 (7.2%)	93 (4.1%)	256 (11.3%)	0.0231
Change of formulation	219 (9.7%)	207 (9.2%)	426 (18.8%)	0.0353
Miscellaneous	260 (11.5%)	797 (35.2%)	1057 (46.7%)	< 0.00001
Total	740 (32.7%)	1522 (67.3%)	2262 (100%)	< 0.00001

^*^Fisher´s exact test (https://www.socscistatistics.com/tests/fisher/default2.aspx)

## Discussion

The study was based on data routinely recorded in the pharmacies. This retrospective study design is believed to reflect how the pharmacist documents interventions in the everyday setting. Most previous studies have been based on prospective collection of data through completing forms by the participating pharmacists, and this may cause a bias towards increased awareness of detecting prescribing errors of potential clinical importance during the study period [2]. However, this retrospective design may have caused underestimation of the number of interventions if the pharmacists have not documented all interventions in a busy everyday setting. In this study all generic substitutions were automatically documented. A similar detection through manually completing forms, would significantly increase the workload of participating pharmacists. The participating community pharmacies belonged to one chain and the results may thus not be representative. All Norwegian pharmacies have, however, implemented a common frame of routines for dispensing prescriptions. This joint focus also includes the outpatient departments of the hospital pharmacies, further minimizing a limitation of not including all pharmacy chains. Another limitation might be comparison of only 4 outpatient departments of hospital pharmacies to 11 community pharmacies. This limitation has been partly compensated by prolonging the study period in the hospital pharmacies to achieve a comparable number of prescriptions in relation to the community pharmacies. The number of pharmacists working in each pharmacy was not reported and it was not possible to estimate workload based on total number of prescriptions dispensed in each pharmacy. The total number of individual pharmacists performing the interventions in the 6 months period of the four hospital pharmacies is, however, likely to be less than the number of pharmacists dispensing prescriptions in the 3 months period of the 11 community pharmacies.

The differences in number of miscellaneous interventions of potential clinical importance in community pharmacies and hospital pharmacies may suggest different routines for documentation of interventions in the two settings. A change from a prescribed strength to half the prescribed strength with double dosing, would be defined as a technical intervention due to drug shortage in this study, unless the pharmacist has given free text information that this intervention was done e.g. due to patient difficulties of swallowing large tablets. In the latter case this intervention was reclassified from a technical intervention to an intervention of potential clinical importance through discussion in the research team. The extensive use of free text fields by the pharmacists in hospital pharmacies, may indicate more focus on recording the reason for an intervention as the patient is still monitored by the hospital physician and a perceived need to ensure effective ongoing communication.

The highest number of interventions belonged to the miscellaneous category, indicating that many interventions of potential clinical importance did not fit into the given categories of the pharmacy computer software. In some of the cases the strength or dose was changed without changing the total daily dose to simplify the administration or to prevent an ADE. Another example was change of dispensed drug from a half tablet to an elderly patient, to an available whole tablet of the same drug at half the strength or a smaller tablet to ease a patient trouble of swallowing large tablets. These are examples of interventions of potential clinical importance which are not due to prescription errors, but rather to enhance patient adherence and thus prevent ADE.

Our study found a twice as high incidence of interventions of potential clinical importance in hospital pharmacies than in community pharmacies. It was not recorded if the prescriber was a hospital physician or a general practitioner. However, the hospital pharmacies dispense a higher percentage of prescriptions from hospital physicians than a community pharmacy. A study undertaken in Norway three years before the introduction of EP, reported more than four times higher incidence of prescribing errors by hospital physicians than general practitioners [2]. This may be due to less experience in outpatient prescribing by doctors at hospitals. A higher incidence of interventions of potential clinical importance in hospital pharmacies, may thus be partly due to a lower EP quality in hospital pharmacies in relation to community pharmacies. On the other hand, the extensive use of free text fields to further explain the nature of an intervention in hospital pharmacies, may suggest an underestimation of documented interventions of potential clinical importance in community pharmacies.

Our study suggests that 0.8% of all dispensed prescriptions in Norwegian pharmacies requires an intervention to correct a prescribing error of potential clinical importance or to prevent an ADE. This compares with an intervention rate of 0.89% per items dispensed in an UK study of clinical interventions by community pharmacies [7]. However, a recent review by Assari et al. of 60 studies, states that there is a very wide variation in medication error and error-related adverse events rates reported in different studies of medication errors in a community setting, reflecting the lack of an international standard of study method and classification of interventions as potential clinical important [10]. As an example, our study did not regard “use as directed” as a prescription error, were other studies categories such a dosing as an error of unclear or omitted information. A similar retrospective study of errors associated with outpatient computerized prescribing systems in an American pharmacy chain, reported a 0.9% incidence of clinical prescribing errors of potential patient harm, and a similar incidence of 4.4% when defining omitted or unclear dosage information as a prescription error [11]. Our study also categorized all generic substitutions as technical interventions, although a study in Switzerland classified a generic substitution as pharmaceutical intervention of potential clinical importance if the patient could not afford the original product [12].

A Norwegian study of pharmaceutical interventions undertaken before the introduction of EP, reported an incidence of 0.68% in pharmaceutical interventions based on clinical prescribing errors [2]. Our finding of a similar incidence of pharmaceutical interventions of potential clinical importance after the introduction of EP, suggest that the main goal of reducing prescription errors leading to ADE with EP has not been met. An US study also showed that the rate of medication errors remained the same after introduction of EP [13]. Another study found that missing information in prescriptions was significantly less after introduction of EP, but the incidence of higher risk errors of incorrect information was more common in EP [14]. This shows that the use of technology through EP has limited effect on prescribing errors of potential clinical importance, and that pharmacist assessing prescriptions to detect prescribing errors is as important today as before the introduction of EP.

Our study implies that the incidence of prescribing errors of potential clinical importance has not been reduced by the introduction of EP. This is in agreement with the results from a study of medication errors in relation to implementing eHealth technologies in Norway from 2015 to 2019 undertaken by the Norwegian Centre for E-health Research [15]. Although the introduction of EP has eliminated formal errors like omissions of patient or prescriber information previously found to occur in 1.5% of prescriptions in Norway before introducing EP, our study shows a high incidence of pharmaceutical interventions of technical nature [2]. This reflects that an EP is a prescription for a single package ID number (Nordic Article Number), of which there may be multiple generic alternatives of different brands and package sizes with other ID numbers. Further our study shows that the pharmacist makes a logistic or pharmacoeconomic intervention on more than 40% of all the prescriptions dispensed. These technical interventions seem to eradicate the aim of introducing EP to reduce pharmacist time spent on formal errors to enable increased time and focus on patient supervision to further prevent ADE. This is also in line with a recent study showing that new prescriptions transmitted by EP required pharmacy-physician office communications fourfold more frequently than faxed prescriptions and nearly twofold more frequently than written prescriptions [16].

It has been estimated that 10% of all hospitalizations of elderly patients are related to an ADE, and 12% of all patient harms in Norwegian hospitals are related to medication errors [3, 15]. The extra burden of medication errors in the Norwegian society is estimated to cause more than 490,000 extra hospital bed-days and resulting in more than 1000 deaths annually [17]. According to The Norwegian Pharmacy Association (Apotekforeningen) the Norwegian pharmacies dispensed a total of 52 million prescriptions in 2018. This study indicating that pharmacists performs an intervention of potential clinical importance in 0.8% of all prescriptions implies that more than 400 000 interventions of potential clinical importance are performed annually in Norwegian pharmacies. A French study of pharmaceutical interventions on clinical outcome and cost avoidance, showed that every Euro invested in the prescription review activity would potentially give 5.09 Euros of saved public health spending [18]. Despite their contribution to public health savings there are presently no economic incentives for pharmacists in Norway to perform interventions unless it is a generic substitution.

## Conclusion

A substantial number of pharmaceutical interventions are performed on prescriptions in Norwegian pharmacies. A potential clinical important intervention is performed in 1 of every 125 prescriptions (0.8%). This result indicates that pharmacists at Norwegian pharmacies prevent more than 400,000 prescription errors of potential clinical importance each year.

## Data Availability

The data from the pharmacy computer system which the study is based on is available.
